# A comparison of conventional high-flow nasal cannula vs the duet high-flow nasal cannula in extubated patients: a randomized crossover trial

**DOI:** 10.1186/s40001-025-02447-0

**Published:** 2025-03-26

**Authors:** Ravindranath Tiruvoipati, Mallikarjuna Ponnapa Reddy, Eldho Paul, Sachin Gupta, Ashwin Subramaniam, David Zhang, Kavi Haji

**Affiliations:** 1 Department of Intensive Care, Peninsula Health, Frankston, VIC 3199 Australia; 2 Division of Medicine, Peninsula Clinical School, Monash University, Frankston, VIC Australia; 3 ANZIC-RC, School of Public Health and Preventive Medicine, Monash University, Melbourne, VIC Australia; 4 Department of Intensive Care, North Canberra Hospital, Canberra, ACT Australia; 5 Department of Anaesthesia and Pain Medicine, Nepean Hospital, Kingswood, NSW Australia; 6 Department of Intensive Care, Dandenong Hospital, Monash Health, Dandenong, VIC Australia; 7 Department of Surgery, University of Melbourne, Melbourne, VIC Australia

**Keywords:** High flow, Nasal cannula, Extubation, Comfort

## Abstract

**Objectives:**

High-flow nasal cannulae (HFNC) are often used for oxygen delivery in intensive care units (ICU). A new configuration HNFC, the Duet HFNC, can deliver higher pressures and potentially improve gas exchange. The clinical performance, safety and patient comfort of these Duet cannula are unclear. Furthermore, there is no data on performance of conventional or Duet HFNC in patients with nasogastric tubes (NG) or between male and female patients. We compared the gas exchange, tolerance, and comfort with conventional and Duet HFNC.

**Research methodology:**

This single-center, open labeled randomized crossover study included invasively ventilated patients who were extubated. Patients were randomized to protocol A (*n* = 25; Duet cannula for 30 min followed by conventional HFNC for 30 min) or protocol B (*n* = 25; conventional followed by Duet HFNC). Subgroup analysis included comparisons between patients with and without a NG, and between male and female patients when receiving Duet and conventional HFNC. During the study period the HFNCs flow rates were set at 40 L/min with the same FiO_2_ standardizing the comparisons for both cannulas.

**Results:**

There was no significant difference in PaO_2_ (86.8 [22.2] vs. 88.9 [23.5]; *p* = 0.38), PaCO_2_ (37.7 [3.9] vs. 37.8 [3.9]; *p* = 0.81) and SaO_2_ (96.2% [2.0] vs. 96.4% [1.8]; *p* = 0.32), comfort and tolerance between the Duet and conventional HFNC. The subgroup analysis did not show a significant difference between these cannulas in patients with or without nasogastric tubes or between males and females.

**Conclusion:**

Duet HFNC is comparable to conventional HFNC. Further studies on higher flow rates and for longer period are warranted to determine the utility of Duet cannula.

*Clinical trial registration:* This trial was registered with ClinicalTrials.gov (Number: NCT06398951) on 3rd May 2024- Retrospectively registered.

## Introduction

Oxygen therapy is one of the common treatments administered to patients in the intensive care unit (ICU) [1]. Several types of oxygen delivery devices are currently available. Currently, oxygen therapy in non-intubated patients may be delivered using several devices. These include conventional high-flow face mask such as Hudson mask, at 10–15 L/min of blended oxygen and air, or low flow nasal cannula and more increasingly high-flow nasal cannula (HFNC). Oxygen delivery via HFNC offers higher oxygen concentration and at a higher flow rate with greater tolerability and comfort than when administered by a high-flow face mask [2]. Oxygen therapy by HFNC has subsequently shown to be useful in both hypoxic and hypercapnic respiratory failure, post-extubation respiratory failure, pre-oxygenation, and in post-operative patients [3–5].

The physiological advantages of HFNC include increased airway pressure, increased end-expiratory lung volume, reduction in dead space ventilation, improvement in the muco-ciliary clearance due to humidification, and overall enhancement of lung compliance and aeration [6–9]. Given these advantages HFNC are considered as primary intervention in patients with hypoxemic respiratory failure to avoid intubation, pre-oxygenation prior to endotracheal intubation, increase ventilator free days as well as prevent reintubation in extubated patients [10–12]. HFNC were also showed to be non-inferior to non-invasive ventilation in patients with acute exacerbation of COPD but had higher rates of the need for intubation as compared to non-invasive ventilation [13].

Recently a new mode of high-flow oxygen therapy called “Duet/Two nare HFNC” (Optiflow^™^ Duet, Fisher and Paykel Healthcare, Auckland, New Zealand) was introduced into clinical practice where the cannula are asymmetrical with different diameters (left cannula larger than the right) [8, 14]. Similar to the conventional HFNC, the Duet HFNC delivers gases at a flow rate range of 10–70 L/min. The difference in the diameters however, could provide additional benefits of different levels of positive pressure, increased carbon dioxide clearance and reduced work of breathing as compared to conventional HFNC [8, 15]. This positive pressure could potentially facilitate enhanced gas exchange in respiratory failure patients, but there is no clinical data to support this. Furthermore, it is unknown if the Duet HFNC are comfortable for patients or have any adverse effects that could be associated with the increased pressures generated by the asymmetric cannula configuration. The increased pressure may promote intolerance and discomfort to patients which could encourage them to open their mouths or even remove the cannulae which in effect could negate the potential benefits of the Duet cannula.

While conventional HFNC are in clinical practice for nearly two decades, there are no studies that compared their performance between male and female patients given the significant differences noted in the nasal cavity configuration and volume between male and female patients [16]. Furthermore several patients in intensive care units and hospitals will have nasal devices such as nasogastric tubes (NGT) and nasopharyngeal temperature probes. The impact of such devices on gas exchange or comfort when using HFNC is unknown.

The aim of our study was to compare the Duet HFNC with conventional HFNC in extubated patients in ICU. Our hypothesis was that Duet HFNC provides equivalent gas exchange as compared to conventional HFNC which are frequently used non-invasive respiratory support in critically ill patients [17].

## Methods

**Trial design:** This was a randomized crossover study of invasively ventilated patients suitable for extubation.

**Aims:** The aims of the study were to:

- Compare Duet HFNC and conventional HFNC for gas exchange (PaO_2_, PaCO_2_, and SaO_2)_, physiological parameters (including heart rate, blood pressure and respiratory rate), tolerance (assessed by the bedside nurse) and comfort (reported by the patient).
- Compare the efficacy of conventional HFNC on gas exchange, physiological parameters tolerance and comfort between males and females and those with and without nasogastric tubes.
- Compare the efficacy of Duet HFNC on gas exchange, physiological parameters tolerance and comfort between males and females and those with and without nasogastric tubes.

**Study setting:** Single-center level III ICU at Frankston Hospital, Victoria, Australia.

**Study period:** The patients were enrolled from 03 March 2023–12 January 2024.

**Patients:** Fifty patients were enrolled in the trial, 25 in each arm. The lack of data on Duet HFNC prior to commencing this study precluded us from performing a power calculation to determine a sample size.

**Fig. 1:**
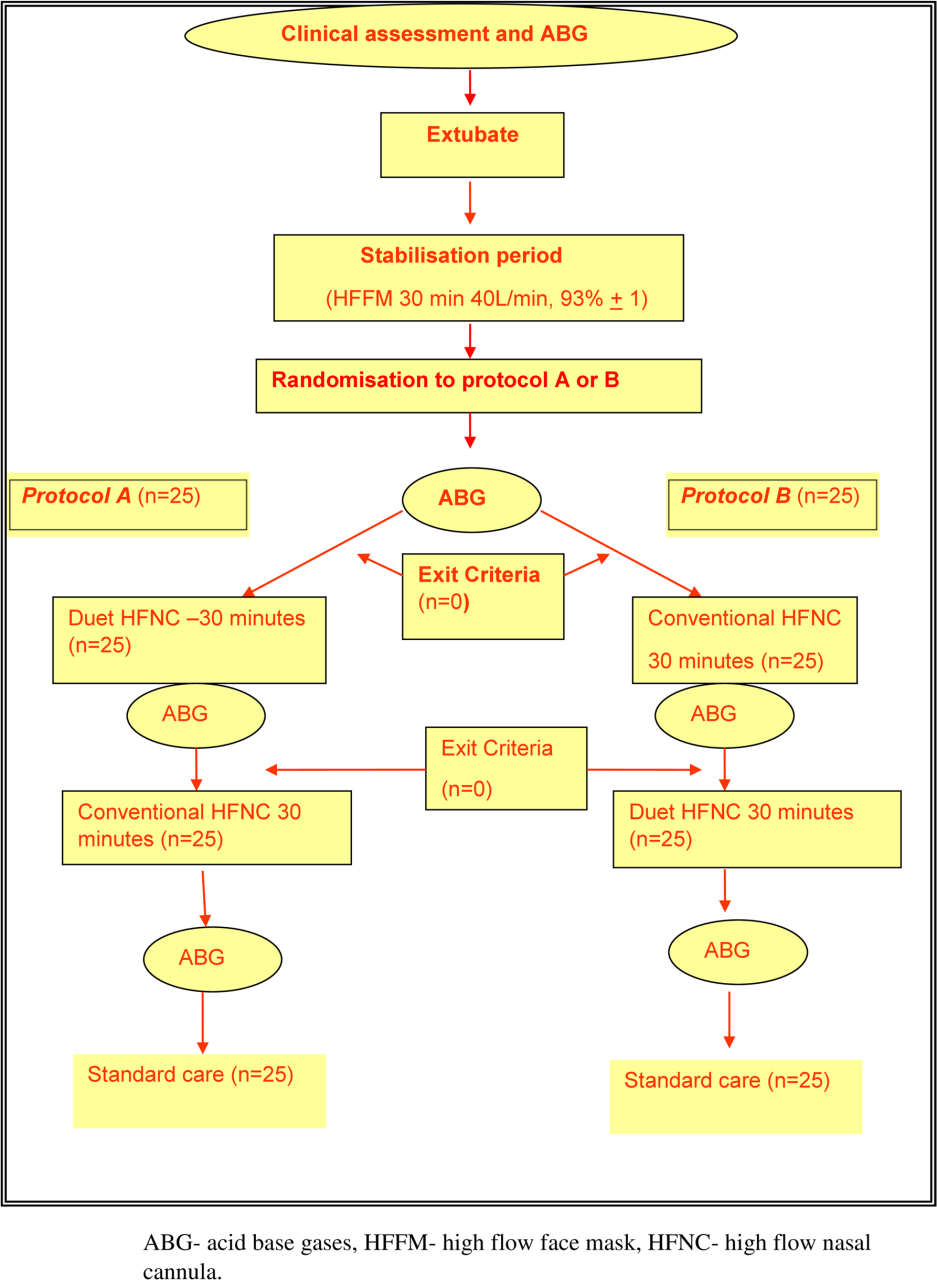
Consort diagram of flow of patients

**Intervention and controls:** Duet HFNC (intervention) were compared with conventional HFNC (Fig. 1). Prior to initiation of the study, Duet HFNC was used in the intensive care unit for 1 month to ensure the device was familiar to the clinicians.

**Inclusion criteria:** Imminently extubated patients were considered suitable for admittance into the trial if their PaO_2_/FiO_2_ ratio was > 150 prior to extubation and had a functional arterial catheter to facilitate arterial blood gas (ABG) sampling.

**Exclusion criteria:** The patients were ineligible if they were: (1) extubated due to withdrawal of therapy or expected to imminently die post–extubation; (2) extubated on to non-invasive ventilation; (3) known epistaxis or postnasal surgery; and (4) confused and unable to tolerate the HFNC or unable to answer the questionnaire.

**Exit criteria:** The following criteria was considered after each ABG to determine whether a patient remains in the trial or is withdrawn from the trial. This was to ensure the safety of the patients while the study was conducted. Withdrawal from the trial was implemented if a patient meets one or more of the following criteria.

Respiratory: (1) oxygen saturation < 90% on any of the HFNC; (2) respiratory rate > 30 breaths per minute; and/ or (3) pH < 7.30.

Cardiovascular: (1) heart rate > 140 beats per minute; and/or (2) systolic blood pressure < 100 mmHg or > 200 mmHg.

Central nervous system: Altered level of consciousness (drop in Glasgow Coma Scale [GCS] by 2 or more).

### Ethics and consent

Ethical approval was obtained from the Human Research Ethics Committee for Clinical Research at Peninsula Health (HREC Reference Number: HREC/88599/PH-2022/ SSA: SSA/88599/PH-2022, Approval Date 28th November 2022; Title: A comparison of conventional high-flow nasal cannula vs two-nare size high-flow nasal cannula: a randomized crossover trial in extubated patients). The study was conducted in accordance with the ethical standards of the responsible committee on human experimentation and with the Helsinki Declaration of 1975 and local regulatory requirements. Consent was obtained either from the patient, or their medical treatment decision-maker or the legal next of kin. No identifiable data was presented in this manuscript ensuing patient confidentiality. The authors assume responsibility for the fidelity of the trial to the protocol and for the accuracy and completeness of the data and analyses.

### Recruitment and randomization

#### Study design (Fig. 1)

Potential patients were clinically assessed for suitability of extubation. An ABG analysis was performed prior to extubation to ensure that the PaO_2_/FiO_2_ was > 150. If suitable, patients were extubated on to high-flow face mask that provided with a high-flow blend of oxygen and air at 40 L/min of an adequate FiO_2_ to ensure an oxygen saturation of 93% ± 1 for 30 min. At the end of this 30-min stabilization, heart rate, blood pressure, respiratory rate, oxygen saturation, and GCS were recorded and another ABG analysis was performed. If remained stable, patients were randomized to either protocol A (Duet HFNC for 30 min followed by the conventional HFNC for the subsequent 30 min) or protocol B with allocation ratio of 1:1(conventional HFNC for 30 min followed by the Duet HFNC for the subsequent 30 min, Fig. 1). During both protocols gas flow rate and FiO_2_ were unchanged from the settings used during the stabilization period. The same physiological parameters, ABG analysis were obtained while the patients were on Duet and conventional HFNC. In addition, comfort score reported by the patient and tolerance score recorded by the bedside nurse were recorded. The 5-point Likert scale was used to assess the comfort and tolerance of patients for both devices as shown below. In this study, only medium size cannulae (Duet and conventional HFNC) were used to maintain consistency. Randomization was performed using sequentially numbered, opaque, and sealed envelopes to maintain allocation concealment.

#### Determining patient’s comfort

Patients provided a comfort score for the Duet HFNC and conventional HFNC prongs using a 5-point Likert scale [2]. This provided the patient’s perspective on how comfortable the devices were.

1. No discomfort,
2. Little bit discomfort,
3. Little more discomfort,
4. Whole lot more discomfort,
5. Worst discomfort.

#### Determining patient’s tolerance

The bedside nurse documented the patient’s tolerance using a 5-point Likert scale [2]. This provided the bedside nurses assessment on the tolerance of these devices by the patients.

0 Patient never removes the apparatus and does not complain.
1 Patient complains about keeping apparatus on.
2 Patient request removal of the apparatus.
3 Removal of the apparatus by the patient once.
4 Removal of multiple times or non-compliance.

### Outcome measures

*Primary outcome:* To compare PaO_2_, PaCO_2_, and SaO_2_ of patients on the Duet HFNC vs conventional HFNC on the same FiO_2_ at 40 L/min flow rate over a 30-min period.

*Secondary outcomes*: To compare the physiological parameters (heart rate, blood pressure, respiratory rate, oxygen saturation) and comfort and tolerance of patients on the Duet vs conventional HFNC.

### Statistical analysis

Sample size was pragmatically determined and *n* = 50 patients was deemed feasible. It was anticipated that this sample size would provide 80% power at the 5% level of significance to show a difference equivalent to 0.4 standard deviation units on a continuous outcome between conventional and Duet HFNC. All analyses were performed with SAS software version 9.4 (SAS Institute, Cary, NC, USA) and SPSS version 22 (IBM SPSS, Armonk, NY). Continuous variables were summarized using mean (SD) or median (IQR) according to data type and distribution. Categorical variables were expressed as counts and proportions. Differences in primary and secondary outcomes between Duet HFNC and conventional HFNC were assessed using paired Student’s t-test or Wilcoxon signed rank test as appropriate with results reported as mean difference (95%-CI) or median difference (95%-CI). Comparisons between pre-specified subgroups (sex, and presence of NG tube) were performed using Student’s t-test for normally distributed continuous variables, Wilcoxon rank-sum test for skewed continuous variables and Chi-square or Fisher's exact test as appropriate for categorical variables. Cohen’s d effect sizes were calculated to determine the magnitude of differences between conventional vs Duet HFNC and reported with 95% CI. All p values reported are two tailed and a *p* < 0.05 indicated statistical significance.

## Results

Fifty patients were included in the study, of which 29 were males (58%). There were no differences in patients’ demographics and physiological data in protocol A and B at randomization as shown in Table 1. The results of primary and secondary outcomes are presented in Table 2. There was no significant difference in the gas exchange between the Duet HFNC and conventional HFNC. The physiological parameters including heart rate, systolic blood pressure, diastolic blood pressure, respiratory rate, comfort score and tolerance score were also comparable between the two devices (Table 2). No patient met the exit criteria that focused on the safety of the devices during the study period.

**Table 1:** Comparison of patients in protocol A and B at randomization

Variable	Protocol A (*n* = 25)	Protocol B (*n* = 25)	*P* value
Age (years) *N* = 50	55.4 (17.2)	62.2 (16.6)	0.16
Sex, *n* (%) *N* = 50			
Male	13 (52%)	16 (64%)	0.39
Female	12 (48%)	9 (36%)	
Diagnosis, *n* (%) *N* = 50			
Medical	19 (76%)	16 (64%)	0.36
Surgical	6 (24%)	9 (36%)	
Number of hours ventilated, median [IQR] *N* = 49	65 [24–107]	65 [18.5–86.5]	0.54
Glasgow Coma Scale *N* = 49	14 (0.88)	14 (0.93)	0.60
APACHE III Score *N* = 49	57.7 (22.6)	62.8 (22.9)	0.43
PaO_2_ (mmHg) *N* = 49	79.2 (15)	87.1 (31.9)	0.27
PaO_2_/FiO_2_ ratio *N* = 48	301 (112)	239 (117)	0.07
PaCO_2_ (mmHg) *N* = 49	37.9 (3.65)	39.6 (3.22)	0.09
pH *N* = 49	7.43 (0.053)	7.41 (0.044)	0.11
Base excess, median (IQR) *N* = 49	0.5 [−1.5 to 3.7]	0.05 [−2.25 to 2.25]	0.65
Oxygen Saturation (%) *N* = 49	95.5 (1.67)	95.9 (2.53)	0.43
Heart rate per minute *N* = 50	84.9 (14.7)	81 (17.8)	0.40
SBP (mmHg) *N* = 50	137 (23.8)	125 (19.4)	0.05
DBP (mmHg) *N* = 50	68.3 (13.7)	61.9 (10.9)	0.07

Data are mean (SD) unless stated otherwise

*APACHE III* Acute Physiology and Chronic Health Evaluation III, *DBP* diastolic blood pressure, *IQR* interquartile range, *SBP* systolic blood pressure, *SD* standard deviation

**Table 2:** Comparison of Duet HFNC Vs HFNC conventional gas exchange (primary and secondary outcomes)

Variable	Two-nare HFNC (*n* = 50)	HFNC conventional (*n* = 50)	Mean difference	95% CI	*P* value	Cohen’s d (95% CI)
Primary outcome						
PaO_2_ (mmHg) *N* = 49	86.82 (22.22)	88.96 (23.45)	−2.14	−6.89 to 2.61	0.38	0.09 (−0.30 to 0.49)
PaCO_2_ (mmHg) *N* = 49	37.73 (3.98)	37.84 (3.97)	−0.10	−0.95 to 0.74	0.81	0.02 (−0.37 to 0.42)
SaO_2_ (%) *N* = 49	96.22 (2.01)	96.49 (1.85)	−0.27	−0.79 to 0.25	0.32	0.14 (−0.26 to 0.54)
Base excess, Median (IQR) *N* = 49	0.7 (−1.1 to 2.6)	0.5 (−1.5 to 2.7)	0.10	−0.11 to 0.31	0.73	0.01 (−0.38 to 0.41)
pH (SD) *N* = 49	7.42 (0.05)	7.42 (0.05)	0.002	−0.01 to 0.01	0.63	0.04 (−0.36 to 0.44)
Secondary outcomes						
Heart rate/min *N* = 49	80.72 (16.46)	81.90 (16.35)	−1.06	−2.57 to 0.45	0.17	0.07 (−0.32 to 0.47)
SBP (mmHg) *N* = 49	133.06 (22.19)	134.57 (21.75)	−1.76	−4.18 to 0.67	0.16	0.07 (−0.33 to 0.46)
DBP (mmHg) *N* = 49	64.88 (12.53)	67.22 (12.62)	−2.41	−4.72 to −0.10	0.05	0.19 (−0.21 to 0.58)
Respiratory rate/min *N* = 49	18.12 (4.13)	18.61 (3.96)	−0.47	−1.48 to 0.54	0.37	0.12 (−0.28 to 0.52)
Comfort score, Median (IQR) *N* = 42	1 (1–2)	1 (1–2)	0	−0.27 to 0.27	0.20	0.23 (−0.19 to 0.66)
Tolerance score, Median (IQR) *N* = 44	0 (0–0)	0 (0–0)	0	NE	0.13	0.42 (0.01 to 0.83)

Data are mean (SD) unless stated otherwise

*SBP* systolic blood pressure, *DBP* diastolic blood pressure, *NE* not estimable

### Subgroup analysis

#### Comparison of duet HFNC between males and female patients

Subgroup analysis was performed to compare the gas exchange, physiological parameters, comfort and tolerance scores between males and females on the Duet and on conventional HFNC at the same FiO_2_ and a 40 L/min flow rate. There were no significant baseline differences at randomization (Table 3). The gas exchange, physiological parameters, comfort, and tolerance scores between male and female patients were comparable between the two groups (Table 4).

**Table 3:** Comparison of blood gasses and physiological variables between male and female patients before randomization

Variable	Male (*n* = 29)	Female (*n* = 21)	*P* value
Age (years) *N* = 50	56.4 (18)	62.1 (15.5)	0.24
Glasgow coma scale *N* = 49	14 (0.99)	14 (0.74)	0.41
APACHE III score *N* = 49	65 (23.6)	54 (20.3)	0.09
PaO_2_ (mmHg) *N* = 49	84.6 (22.9)	80.9 (27.8)	0.62
PaO_2_/FiO_2_ ratio *N* = 49	250 (116)	298 (117)	0.16
PaCO_2_ (mmHg) *N* = 49	38.7 (3.65)	38.8 (3.4)	0.92
pH *N* = 49	7.42 (0.054)	7.42 (0.042)	0.81
Base excess, median (IQR) *N* = 49	0.5 [−1.4 to 2.9]	0.15 [−1.75 to 3.2]	0.97
SaO_2_ (%) *N* = 49	95.8 (2.14)	95.6 (2.15)	0.74
Heart rate/min *N* = 50	83.9 (18.2)	81.7 (13.5)	0.64
SBP (mmHg) *N* = 50	135 (24.1)	126 (19.3)	0.2
DBP (mmHg) *N* = 50	67.5 (13)	61.8 (11.6)	0.12

Data are mean (SD) unless stated otherwise

*APACHE III* Acute Physiology and Chronic Health Evaluation III, *DBP* diastolic blood pressure, *IQR* interquartile range, *SBP* systolic blood pressure, *SD* standard deviation

**Table 4:** Comparison of Duet HFNP between male patients and female patients

Variable	Males (*n* = 29)	Females (*n* = 21)	Mean difference	95% CI	*P* value	Cohen’s d (95% CI)
Gas exchange						
PaO_2_ (mmHg) *N* = 49	85.9 (19)	88.2 (26.7)	−2.34	−15.11 to 10.44	0.72	0.10 (−0.47 to 0.67)
PaCO_2_ (mmHg) *N* = 49	37.7 (4.47)	37.9 (3.27)	−0.19	−2.49 to 2.10	0.87	0.05 (−0.52 to 0.62)
SaO_2_ (%) *N* = 49	96.4 (1.56)	96 (2.55)	0.38	−0.77 to1.53	0.52	0.19 (−0.38 to 0.76)
Base excess, median (IQR) *N* = 49	0.9 (−1.1 to 2.4)	0.25 (−1.3 to 3)	0.60	−2.14 to 3.34	0.96	0.04 (−0.53 to 0.61)
pH *N* = 49	7.42 (0.056)	7.43 (0.043)	0.00	−0.03 to 0.03	0.94	0.20 (−0.37 to 0.77)
Physiological data						
Heart rate/min *N* = 49	81.8 (16.7)	79.2 (16.4)	2.64	−6.67 to 11.95	0.58	0.16 (−0.41 to 0.72)
SBP (mmHg) *N* = 49	137 (23.2)	128 (20.3)	8.48	−3.88 to 20.84	0.19	0.41 (−0.15 to 0.98)
DBP (mmHg) *N* = 49	67.4(10.8)	61.4 (14.2)	5.95	−0.96 to 12.86	0.10	0.48 (−0.09 to 1.04)
Respiratory rate/min *N* = 49	18.9 (3.74)	17 (4.5)	1.85	−0.44 to 4.14	0.12	0.46 (−0.11 to 1.03
Comfort score, Median (IQR) *N* = 42	1 (1–2)	1 (1–2)	0	−0.82 to 0.82	0.98	0.08 (−0.53 to 0.70)
Tolerance score, Median (IQR) *N* = 48	0 (0–0)	0 (0–1)	0	NE	0.19	0.38 (−0.84 to 84)

Data are mean (SD) unless stated otherwise. *SBP* systolic blood pressure, *DBP* diastolic blood pressure, *NE* no estimate

#### Comparison of duet HFNC between patients with and without nasogastric tubes

Nasogastric tubes were present in 39 (78%) patients. There were no baseline differences in age, sex, GCS between patients with or without nasogastric tubes at the time of randomization (Table 5). The APACHE III scores were higher in patients who had NG tubes (Table 5). On a gas flow of 40 L/min and the same FiO_2_, there were no differences in gas exchange comfort, physiological variables or tolerance with the use of Duet HFNC (Table 6).

**Table 5:** Comparison of blood gasses and physiological variables between patients with and without NGT before randomization

Variable	NG tube (*n* = 39)	No NG tube (*n* = 11)	*P* value
Age (years)	60.4 (15.9)	53.1 (20.4)	0.21
Sex, *n* (%)			
Male	27 (69.2)	2 (18.2)	0.004
Female	12 (30.8)	9 (81.8)	
Glasgow coma scale *N* = 49	14 [14, 15]	15 [14, 15]	0.07
APACHE III Score	65.3 (21.8)	42.9 (16.9)	0.003
PaO_2_ (mmHg) *N* = 49	80.5 (24)	91.9 (26.7)	0.18
PCO_2_ (mmHg) *N* = 49	39.1 (3.76)	37.5 (2.25)	0.2
pH *N* = 49	7.42 (0.052)	7.41 (0.04)	0.37
Base excess, Median (IQR) *N* = 49	0.75 [−0.4 to −4.1]	−1.5 [−3.1 to −1.7]	0.18
SaO_2_ (%) *N* = 49	95.5 (2.15)	96.2 (2.06)	0.36
Heart rate/min *N* = 49	82.5 (17)	84.5 (13.8)	0.72
SBP (mmHg) *N* = 49	132 (24.2)	129 (15)	0.71
DBP (mmHg) *N* = 49	65.7 (12.2)	62.9 (14.6)	0.52

Data are mean (SD) unless stated otherwise

*APACHE III* Acute Physiology and Chronic Health Evaluation III, *DBP* diastolic blood pressure, *IQR* interquartile range, *SBP* systolic blood pressure, *SD* standard deviation

**Table 6:** Comparison of Duet HFNC between patients who had NGT vs those who did not have NGT

Variable	NG present (*n* = 38)	NG absent (*n* = 11)	Mean difference	95% CI	*P* value	Cohen’s d (95% CI)
Gas exchange						
PaO_2_ (mmHg) *N* = 49	84.6 (18.9)	94.4 (31.2)	−9.73	−24.54 to 5.08	0.20	0.38 (−0.30 to 1.06)
PaCO_2_ (mmHg) *N* = 49	37.9 (4.23)	37.2 (3.09)	0.71	−1.98 to 3.41	0.61	0.19 (−0.48 to 0.86)
SaO_2_ (%) *N* = 49	96.1 (1.99)	96.7 (2.09)	−0.62	−1.97 to 0.73	0.37	0.29 (−0.38 to 0.97
Base excess, Median (IQR) *N* = 49	0.95 (−0.9 to 3.8)	−1.1 (−2.7 to 1.7)	2.0	−0.93 to 4.93	0.14	0.59 (−0.09 to 1.27)
pH *N* = 49	7.43 (0.05)	7.4 (0.05)	0.03	−0.01 to 0.06	0.14	0.60 (−0.08 to 1.28)
Physiological data						
Heart rate/min *N* = 49	80.4 (16)	82 (18.7)	−1.64	−12.76 to 9.48	0.77	0.09 (−0.58 to 0.76)
SBP (mmHg) *N* = 49	134 (24.3)	128 (11.9)	6.37	−8.52 to 21.26	0.41	0.31 (−0.36 to 0.99)
DBP (mmHg) *N* = 49	65.2 (12.3)	63.9 (13.8)	1.24	−7.22 to 9.71	0.77	0.10 (−0.57 to 0.77)
Respiratory rate/min, mean (SD) *N* = 49	18.3 (4.11)	17.4 (4.32)	0.97	−1.81 to 3.75	0.50	0.21 (−0.46 to 0.88)
Comfort score, median (IQR) *N* = 42	1 (1–2)	1.5 (1–2.5)	0	−1.28 to 1.28	0.57	0.23 (−0.54 to 1.00)
Tolerance score, median (IQR) *N* = 48	0 (0–0)	0 (0–2)	0	NE	0.11	0.58 (−0.68 to 0.68)

Data are mean (SD) unless stated otherwise

*APACHE III* Acute Physiology and Chronic Health Evaluation III, *DBP* diastolic blood pressure, *IQR* interquartile range, *SBP* systolic blood pressure, *SD* standard deviation

#### Comparison of conventional HFNC between males and female patients

No significant difference was noted on gas exchange or comfort and tolerance score between male and female patients with conventional HFNC on the same FiO_2_ at a flow rate of 40 L/min (Table 7).

**Table 7:** Comparison of conventional HFNC between male patients and female patients

Variable	Males (*n* = 29)	Females (*n* = 21)	Mean difference	95% CI	*P* value	Cohen’s d (95% CI)
Gas exchange						
PaO_2_ (mmHg) *N* = 49	90.4 (24.3)	86.8 (22.6)	3.65	−9.81 to 17.11	0.60	015 (−0.42 to 0.72)
PaCO_2_ (mmHg) *N* = 49	37.8 (4.12)	37.9 (3.83)	−0.02	−2.31 to 2.26	0.99	0.03 (−0.54 to 0.59)
SaO_2_ (%) *N* = 49	96.7 (1.93)	96.1 (1.7)	0.59	−0.46 to 1.64	0.27	0.33 (−0.24 to 0.90)
Base excess, median (IQR) *N* = 49	0.6 (−1.3 to 2.5)	0.05 (−2.05 to 2.85)	−0.50	−3.19 to 2.19	0.87	0.01 (−0.56 to 0.58)
pH *N* = 49	7.42 (0.06)	7.42 (0.04)	0	−0.03 to 0.03	0.91	0 (−0.57 to 0.57)
Physiological data						
Heart rate/min *N* = 49	82.9 (17.6)	80.4 (14.6)	2.53	−6.85 to 11.92	0.60	0.15 (−0.42 to 0.73)
SBP (mmHg) *N* = 49	136 (21.4)	132 (22.6)	3.84	−8.63 to 16.31	0.55	0.18 (−0.39 to 0.75)
DBP (mmHg) *N* = 49	69.1 (11.9)	64.5 (13.4)	4.69	−2.45 to 11.83	0.21	0.36 (−0.21 to 0.94)
Respiratory rate/min *N* = 49	19.1 (3.61)	17.9 (4.39)	1.29	−0.96 to 3.54	0.27	0.30 (−0.27 to 0.87)
Comfort score, median (IQR) *N* = 42	1 (1–2)	1 (1–2)	0	−0.55 to 0.55	0.75	0.12 (−0.50 to 0.74)
Tolerance score, median (IQR) *N* = 45	0 (0–0)	0 (0–0)	0	NE	0.69	0.14 (−0.60 to 0.60)

Data are mean (SD) unless stated otherwise

*SBP* systolic blood pressure, *DBP* diastolic blood pressure, *NE* not estimable

#### Comparison of conventional HFNC between patients with and without nasogastric tubes

There was a trend towards statistical significance in PaO2 when patients with and without nasogastric tubes were comparted with patients without NG tubes having a higher PaO_2_ (101 (33.3) vs. 85.5 (19); mean difference 15.4; *p* = 0.05, Cohen’s d 0.57 (95% CI (−0.11 to 1.25)). There were no other differences in the gas exchange (PaCO_2_ or SaO_2_) or the comfort and tolerance scores (Table 8).

**Table 8:** Comparison of conventional HFNC between patients with NGT and without NGT

Variable	NG present (*n* = 38)	NG absent (*n* = 11)	Mean difference	95% CI	*P* value	Cohen’s d (95% CI)
Gas exchange						
PaO_2_ (mmHg) *N* = 49	85.5 (19)	101 (33.3)	−15.41	−30.69 to 0.13	0.05	0.57 (−0.11 to 1.25)
PaCO_2_ (mmHg) *N* = 49	38.1 (4.18)	37 (3.13)	1.08	−1.59 to 3.75	0.43	0.30 (−0.38 to 0.97)
SaO_2_ (%) *N* = 49	96.3 (1.87)	97.1 (1.71)	−0.75	−1.98 to 0.49	0.24	0.45 (−0.23 to 1.12)
Base excess, median (IQR) *N* = 49	0.75 (−1.3 to 3.4)	−1.4 (−2.8 to 0.6)	2.0	−0.55 to 4.55	0.08	0.69 (0 to 1.37)
pH *N* = 49	7.43 (0.051)	7.4 (0.05)	0.03	0.0 to 0.06	0.10	0.59 (−0.09 to 1.28)
Physiological data						
Heart rate/min *N* = 49	81.7 (16.6)	82.7 (16.1)	−1.07	−12.15 to 10.01	0.85	0.06 (−0.61 to 0.73)
SBP (mmHg) *N* = 49	136 (23.9)	130 (11.8)	5.43	−9.24 to 20.09	0.47	0.32 (−0.36 to 0.99)
DBP (mmHg) *N* = 49	68.3 (12.5)	63.6 (12.9)	4.63	−3.83 to 13.08	0.29	0.37 (−0.31 to 1.05)
Respiratory rate/min *N* = 49	19 (4.08)	17.3 (3.32)	1.73	−0.91 to 4.37	0.21	0.46 (−0.22 to 1.13)
Comfort score, median (IQR) *N* = 42	1 (1–2)	1 (1–2.5)	0	−1.28 to 1.28	0.63	0.28 (−0.49 to 1.05)
Tolerance score, median (IQR) *N* = 45	0 (0–0)	0 (0–0)	0	NE	0.32	0.49 (−0.74 to 0.74)

Data are mean (SD) unless stated otherwise

*SBP* systolic blood pressure, *DBP* diastolic blood pressure, *NE* not estimable

## Discussion

### Key findings

This randomized controlled crossover study showed that the Duet HFNC were comparable to the conventional HFNC for gas exchange as well as physiological parameters, comfort, and tolerance. The subgroup analyses also demonstrated no differences in gas exchange, physiological parameters, comfort, and tolerance with Duet HFNC between males and females or in patients with and without nasogastric tubes. However, with conventional HFNC, there was a trend towards better oxygenation in patients without nasogastric tubes.

### Relationship with previous studies

Duet HFNC was first tested in an airway model, which suggested that differences in the nasal cannula diameters would result in differences in the prong/nare ratio. An increase in flow rate as well as an increase in the prong/nare area ratio could increase positive airway pressure and enhance oxygenation as well as ventilation when compared with conventional HFNP [15]. This laboratory study reflects a mouth closed position which is not consistent in clinical practice. Laboratory studies [18] also have shown that a smaller cannula outlet may reduce the clearance time and increase airway pressures at 30, 60, and 90 L/min. The clinical implications of these are unknown. Notably, the compliance of every patient in maintaining a closed mouth using these cannulae would not be achievable. Furthermore, there were no laboratory studies that evaluated the performance of these devices with nasal devices present. Hence the need for clinical studies to investigate the performance of these devices in gas exchange, safety and comfort for patients when these devices are used.

There is limited clinical data on Duet HFNC. In a randomized sequence, Slobod et al. [19], investigated the asymmetric HFNC on 10 hypoxemic patients in the ICU at 40 L/min for 15 min followed by 60 L/min for 15 min while continuously monitoring the patients with esophageal manometry and electrical impedance tomography. While the work of breathing and minute ventilation were reduced at a flow rate of 60 L/min, there was no significant improvement in gas exchange. This study did not investigate the patient tolerance or comfort with Duet HFNC. Boscolo et al., investigated Duet HFNC at a flow rate of 60 L/min in a randomized physiological crossover study of 20 patients with hypoxemic acute respiratory failure in the ICU. The study found no differences in gas exchange, dyspnea as compared to conventional HFNC [20]. In this study, patient comfort was slightly improved when compared to the conventional HFNC, however, our study did not confirm this finding. It is possible that the difference noted in the comfort may in the Boscolo study could be due to different sizes of the cannula used as well as varying flow rates between 50 and 60 L/min. In our study, we used medium cannula and a consistent flow of 40 L/min to ensure the comparisons are more robust.

None of these two studies investigated the efficacy of Duet or conventional HFNC in the presence of nasal devices such as nasogastric tube. In our study NGT was seen in 78% of patients. There was trend to better oxygenation with conventional HFNC in patients without nasogastric tubes (Table 8). This may likely be due to the impact of nasogastric tubes on oxygen delivery when non-invasive devices such as HFNC and oxygen delivery masks are used for oxygenation. This needs to be assessed in future studies that are larger and sufficiently powered due to the frequent presence of nasogastric tubes in critically ill patients.

Our study further explored if there were differences between male and female patients given the known morphological variations in the nasal cavities between both sexes. We did not find a significant difference either with gas exchange or comfort. Larger studies are required to confirm these findings, both for conventional and Duet HFNC.

The duration of study period in our study was short and hence we did not collect data on adverse events such as mucosal injuries and pressure ulcers that may be associated with these devices. Such data are important to establish the safety profile for duet HFNC.

### Study implications and future directions

The key implication of our findings is that in uncomplicated extubation, the Duet HFNC are as efficacious as the conventional HFNC with regard to gas exchange and the physiological parameters. In addition, the asymmetric design of the HFNC interface did not add any discomfort and was tolerated by the patients. More importantly there were no safety issues with no patient meeting the exit criteria. Therefore, the Duet HFNC can be used as an alternative device to the conventional HFNC. Our study showed a trend towards better oxygenation in those patients without nasogastric tubes with conventional HFNC having higher PaO2 (101 (33.3) vs. 85.5 (19); mean difference 15.4; *p* = 0.05, Cohen’s d 0.57 (95% CI (−0.11 to 1.25)). Therefore patients who are at particular risk of hypoxemia and need for reintubation with nasogastric tubes need more careful assessment on the need for higher oxygen delivery of the ongoing need for nasogastric tube.

However, conventional HFNC are known to be associated with failure of treatment [13] with escalation to non-invasive ventilation and delayed intubation. Such delated intubation was known to be associated with increased risk of mortality [21]. Increased efficiency of ventilation and enhanced carbon dioxide clearance was demonstrated with duet HFNC as compared to conventional HFNC [19]. The implication of this increased ventilation efficiency on treatment failure remains to be evaluated. Our study did not specifically assess patients with hypercapnic respiratory failure. Randomized controlled trials on duet HFNP are required for evaluation of the efficacy of these devices in hypercapnic respiratory failure.

Our study did not explore the role of the Duet HFNC in minimizing reintubation, improving long-term comfort, survival, managing hypercapnic respiratory failure, duration of ICU stay, survival benefits or using it as an alternative therapy to non-invasive ventilation when compared to the conventional HFNC device especially at higher (> 40 L/min) flow rate. Future studies are required to further assess the value of Duet HFNC especially in those with hypercapnic respiratory failure as none of the current studies have specifically investigated hypercapnic respiratory failure.

### Strengths and limitations

#### Strengths

Our study is the largest randomized crossover trial published so far, providing insights into the clinical efficacy and safety profile of Duet HFNC device. It is also the first study to provide a comparison of Duet HFNC and conventional HFNC between both sexes and based on the presence or absence of a nasogastric tube.

#### Limitations

This study had limitations. Our study duration was 30 min for each intervention and may not reflect longer term performance. The comfort and tolerance also was assessed at 30-min duration. This may have changed if the study was continued for longer than 30 min. We have used medium-sized nasal candidates for all patients, and this may not have provided the optimal benefits based on the optimal prong/nare ratio. We have not assessed the changes in gas exchange or other physiological variables at flows other than 40 L/min. The sample size of the subgroups including male and female patients as well as those with and without NG tubes were perhaps not powered to detect a difference due to type II error as well as the influence of potential confounders such as nare-to-cannula ratio [8]. The study was submitted for registration to clinical trials registry (ClinicalTrials.gov) on the 24 March 2024 but was formally registered on 3 May 2024. The delay in registration was due to administrative and logistical reasons. While this makes the registration retrospective, the methodology reported in this manuscript fully aligns with registered trial protocol.

## Conclusions

In uncomplicated extubation in the ICU, the Duet HFNC is comparable to the conventional HFNC device in gas exchange and physiological parameters and is as well tolerated as conventional HFNC.

## Data Availability

No datasets were generated or analysed during the current study.

## Abbreviations

ABG: Arterial blood gas
HFNC: High-flow nasal cannulae
HREC: Human Research Ethics Committee for Clinical Research
ICU: Intensive care unit
NG: Nasogastric tubes
PaCO_2_: Partial pressure of carbon dioxide in arterial blood
PaO_2_: Partial pressure of oxygen in arterial blood
SaO2: Oxygen saturation in arterial blood
